# A grasp point generation algorithm for waste handling based on a generative reasoning network

**DOI:** 10.1371/journal.pone.0349864

**Published:** 2026-05-29

**Authors:** Xiao Xiao, Deyu Liu, Hai Qin

**Affiliations:** 1 School of Electrical Engineering, Hunan Industry Polytechnic, Changsha, Hunan Province, China; 2 School of Electronic Information, Hunan First Normal University, Changsha, Hunan Province, China; 3 Key Laboratory of Hunan Province for 3D Scene Visualization and Intelligence Education, Changsha, Hunan Province, China; Instituto Politecnico Nacional, MEXICO

## Abstract

In the process of urban kitchen waste sorting, robots often encounter issues such as slipping and empty grabs when attempting to grasp dirty waste objects like plastic bottles and glass bottles. This paper proposes a garbage grasping framework based on the Channel Exchange Generative Residual Inference Network (CE-GR-NET), which synthesizes optimal grasping trajectories through the fusion of hierarchical visual features. The object detection network identifies and locates recyclable bottles among solid waste, while CE-GR-NET uses RGB and depth images to generate grasping points for plastic recyclable bottles. Experimental results show that, on the Cornell Grasping Dataset, the proposed method achieves good inference performance and fast inference speed in single-object solid waste scenarios, ultimately generating grasping boxes for plastic recyclable bottles in RGB images. On the self-constructed multi-source urban kitchen waste images, the proposed method generates grasping boxes for targets and regresses the corresponding object categories simultaneously, achieving an image-based model accuracy of 96.03%, an object-based model accuracy of 94.40%, and a grasping object classification accuracy of 97.87%.

## Introduction

Waste management is a global challenge, with hundreds of millions of tons of waste generated daily worldwide, requiring significant resources and costs for proper handling. According to current data and projections, from 2020 to 2050, municipal solid waste is expected to emit approximately 32–35 billion tons of carbon dioxide equivalent greenhouse gases [1,2]. Due to rapid urban population growth and the limited capacity of urban environments, municipal solid waste (MSW) often cannot be properly managed, leading to increasingly serious environmental problems. This poses a significant obstacle to sustainable development. Among urban MSW, there are recyclable components such as plastic bottles, aluminum cans, and Tetra Paks. Waste recycling plays a vital role in reducing emissions, creating value, lowering operational costs, and generating both social and economic benefits. Currently, manual waste sorting faces challenges such as high labor intensity and low efficiency. In addition, traditional image recognition algorithms often rely on limited and homogeneous datasets, which are expensive to construct and lack generalizability. Consequently, the stochastic nature of waste orientation necessitates a more robust framework for pose estimation and grasping accuracy [3].

In recent years, with the rapid development of deep learning techniques, semi-supervised learning has emerged as an effective approach to address challenges such as the high cost of data annotation and the scarcity of labeled data. Among various semi-supervised learning methods, generative models [4] play a significant role by combining generative and discriminative paradigms. These models leverage both labeled and unlabeled data to enhance model performance while generating realistic data samples.In practical applications such as waste classification, grasp point generation is a critical task that can significantly improve the efficiency of robotic waste sorting. To address this problem, Kumra et al [5]. proposed GR-ConvNet (Generative Residual Convolutional Neural Network), which integrates image analysis and robotic control. Through deep learning, GR-ConvNet is capable of generating accurate grasp rectangles from images, providing essential support for robotic systems to grasp waste objects in complex environments. This semi-supervised generative model learns from unlabeled waste images and generates precise grasp frames.

Urban kitchen waste, in addition to biodegradable biomass, often contains a large proportion of other household waste such as plastics, glass, fibers, and metals. Among these, “bottle-like” waste—including plastic bottles, Tetra Paks, aluminum cans, and glass bottles—tends to have relatively intact shapes and visually similar appearances. These items not only require high detection precision but also possess high recycling value. This paper focuses on bottle-like waste as the primary research object and explores grasp rectangle generation using visual methods.

Currently, most research on grasp inference does not involve the classification or identification of the target objects. In this work, we propose CE-GR-NET, a grasp rectangle generation algorithm based on a semi-supervised generative reasoning network. Built upon GR-ConvNet, the proposed model integrates a channel-exchange module and combines object detection and grasp inference networks. This enables the system to perform targeted grasp inference on recyclable objects such as plastic bottles with higher purposefulness and improved sorting efficiency.

## Related work

Recent research has made considerable progress in the recognition and classification of waste images [6]. Tachwali et al. proposed a classification method based on thermal imaging, utilizing Support Vector Machines (SVM) to detect three different types of recyclable items from thermal images. In addition, a decision tree was employed to classify plastic bottles based on chemical composition and color, achieving an accuracy of 83.48%. Currently, deep learning has become the dominant approach in waste classification. Mao et al. applied a Genetic Algorithm (GA) to optimize the fully connected layers of the DenseNet121 model on the TrashNet dataset [7], achieving a highest classification accuracy of 99.6%. Fulton et al. proposed a two-stage waste detection system [8], which offers high accuracy in both localization and recognition. However, the inference speed of two-stage methods is slower compared to one-stage methods, making them less suitable for real-time grasping applications.

In robotic grasp detection, systems can generally be categorized into two types based on the application scenario: 2D planar grasping and 6-DOF (Degrees of Freedom) spatial grasping. In 6-DOF grasping, the robotic arm can move along the x, y, and z axes and rotate about these three axes, allowing full spatial manipulation. In contrast, 2D planar grasping involves top-down grasping from a fixed angle, with the gripper oriented vertically relative to a planar surface. The grasp configuration is simplified from 6D to 3D: 2D planar position plus 1D rotation. When dealing with solid waste, the robotic gripper typically operates above the recycling surface, making it a 2D planar grasping scenario. Existing grasp detection techniques are primarily divided into two categories: analytical methods and data-driven methods [9,10]. 1) Analytical methods rely on hard-coded rules, determining grasp points through kinematic, dynamic, and force analysis of object geometry and pose. These methods often require satisfying numerous constraints, resulting in high computational complexity and low efficiency. They are also less robust to pose estimation errors or external disturbances, which can lead to deviations between the end-effector’s actual and expected positions, causing task space drift. 2) Data-driven methods, on the other hand, are based on machine learning, learning grasp configurations from large datasets of grasping experiences. In real-world scenarios with many previously unseen objects, data-driven methods are more adaptable and effective for grasp reasoning. In recent years, deep learning-based data-driven grasp detection methods have gained widespread adoption [11]. In recent years, deep learning-based data-driven grasp detection methods have gained widespread adoption [11]. Recent studies have also shown that ensemble neural network strategies are effective in identifying optimal features from high-dimensional data, supporting the use of hybrid architectures for robust feature learning in complex environments [12]. These methods can be further divided into discriminative approaches [13] and generative approaches [14]. Discriminative models typically offer higher accuracy, as they evaluate and rank candidate grasps; however, their efficiency is relatively low due to the computational cost of this filtering process. Generative models, in contrast, are more efficient and better suited to real-time applications. In robotic grasp detection tasks, both accuracy and efficiency are crucial performance metrics.

Regarding specific grasp detection frameworks, Satish et al. [15] introduced FC-GQ-CNN, a fully convolutional Grasp Quality Convolutional Neural Network for robust grasp prediction. With a data collection strategy and a synthetic training environment, the model improves grasp quality estimation, enhancing the reliability and efficiency of robotic grasping. L. Antanas et al. introduced a probabilistic logic framework [15] that integrates high-level reasoning (e.g., object hypotheses, categories, and task-based information) with low-level perception based on visual shape features. This method performed well in complex kitchen scenarios. D. Morrison et al. proposed a generative grasping CNN architecture [16], which generates grasp poses directly from depth images in a pixel-wise manner. This approach reduces the drawbacks of discrete sampling and computational complexity, enabling more efficient and accurate grasp inference.

## Grasp detection dataset and system

### Grasp detection dataset

RGB and depth images are the most commonly used data types in grasp inference tasks. Two well-known robotic grasp detection datasets, Cornell [17] and Jacquard [18], are constructed based on these modalities. Although the Jacquard dataset is larger in scale, it is also more difficult to acquire and requires higher training costs. Therefore, in this study, we choose the Cornell grasping dataset and enhance it to better suit our task. Existing studies on grasp detection can be categorized into three types based on the input image modalities: those using RGB-only, depth-only, and RGB-D fused image data. Prior research has demonstrated [5,19] that RGB-D fusion provides greater advantages for grasp inference compared to using RGB or depth data alone, offering improved accuracy and robustness.

**Fig 1:**
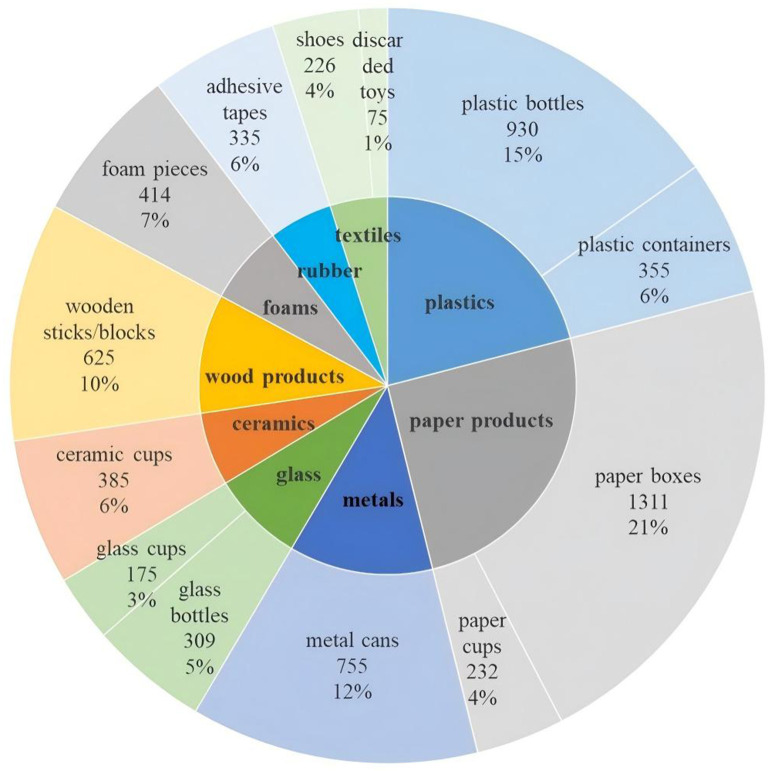
Category-wise distribution of solid waste types in the annotated multi-source image dataset for kitchen waste grasp point generation.

To address the challenges of robotic waste sorting in urban environments, we constructed a multi-source image dataset for grasp point generation with object category labels, specifically targeting urban kitchen waste. As illustrated in Fig 1, the dataset includes nine common categories of kitchen waste impurities and recyclable solid waste items. It consists of 2,161 RGB images and their corresponding 2,161 depth images. The solid waste items in the images are divided into nine major categories and thirteen subcategories, enabling the model to learn both grasp affordances and category-aware representations for more targeted grasp planning.

**Fig 2:**
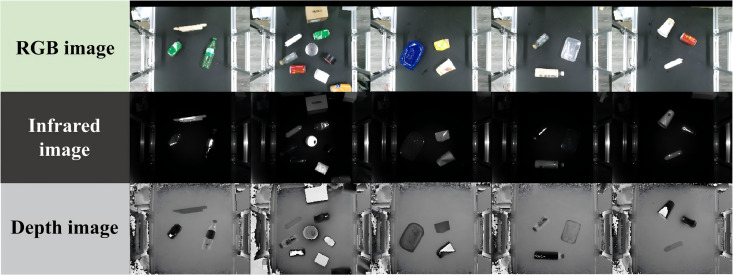
Sample images from the annotated multi-source kitchen waste dataset for grasp point generation.

The RGB, infrared spectral, and depth image samples from our constructed multi-source kitchen waste image dataset are illustrated in Fig 2. The dataset includes a wide range of solid waste categories with the following instance counts: 930 plastic bottles, 355 plastic containers, 1311 paper boxes, 232 paper cups, 755 metal cans, 309 glass bottles, 175 glass cups, 385 ceramic cups, 625 wooden sticks/blocks, 414 foam pieces, 335 adhesive tapes, 226 shoes, and 75 discarded toys. All images have a fixed resolution of 500 × 380 pixels, and more than 10,000 grasp points have been manually annotated across the dataset. Prior to training the grasp detection network, each image undergoes a preprocessing pipeline that includes cropping, resizing, and normalization. The final input image size is standardized to 224 × 224 pixels, ensuring compatibility with the network architecture and training stability. The dataset used in this study is available at: https://github.com/seedahi/AGPGA-data.

### Grasp detection system

**Fig 3:**
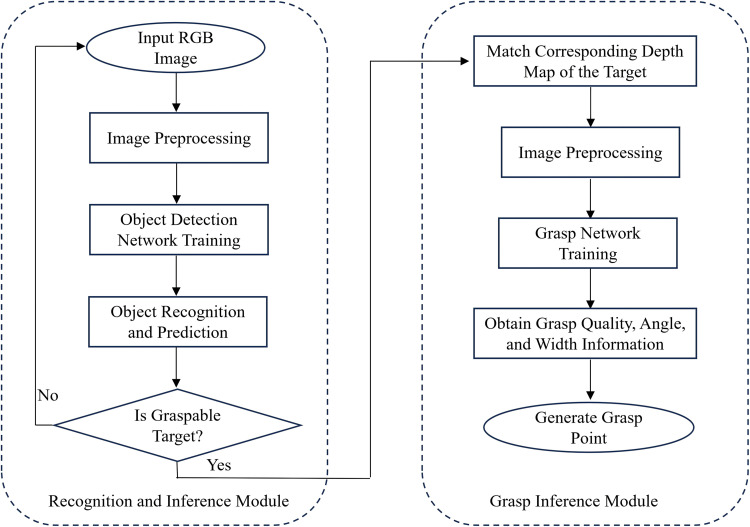
Overall system architecture of the object recognition and grasp inference process.

The vision-based grasp point generation system for plastic bottle recycling comprises two primary components: an object recognition and reasoning module and a grasp inference module, as illustrated in Fig 3. The recognition module is responsible for detecting the presence of target objects, specifically plastic recyclable bottles. Once such objects are detected, the grasp inference module generates feasible paired grasp points for the identified targets. An RGB image of solid waste is first input into the object detection module. If a plastic bottle is detected within the image, the system performs data matching and forwards the RGB image along with its corresponding depth image to the grasp inference module. This module then generates paired grasp points at the pixel level, and computes a grasp quality map, grasp angle map, and gripper width map for each grasp candidate. Based on these maps, the system selects the optimal grasp point and generates the corresponding grasp rectangle. Both the object recognition and grasp inference modules operate at millisecond-level inference speeds. The recognition module processes a single image in approximately 28 ms, while the grasp inference module takes about 200 ms per inference. When deployed on the target development platform, both modules experience an increase in latency, with the average total inference time reaching 500 ms. However, considering the physical operation speed of the robotic manipulator in practical scenarios, the system is still capable of achieving real-time performance for robotic grasping tasks.

In the object recognition and reasoning module, the input stage applies Mosaic data augmentation [20], adaptive image scaling, and anchor box optimization to preprocess the input images. The processed images are then fed into the backbone network, which consists of Focus and Cross Stage Partial (CSP) structures for effective feature extraction. These features are utilized in subsequent processing stages. The Neck component incorporates a Feature Pyramid Network (FPN) and a Path Aggregation Network (PAN) to better fuse the hierarchical features extracted by the backbone. By performing multi-scale downsampling and feature aggregation across different resolutions, the network enhances its ability to detect objects of various sizes, improving recognition performance for targets at different scales. The prediction head generates detection outputs by utilizing anchor boxes on the feature maps to propose potential object locations. These anchors are then used to produce final output vectors consisting of class probabilities, confidence scores, and bounding box coordinates. The grasp inference module consists of three main stages:

1. Preprocessing: Input images are cropped, resized, and normalized. Each RGB image is matched with its corresponding depth map to obtain depth representations [16]. The network accepts 224 × 224 pixel inputs and supports n-channel configurations, allowing for flexible input modalities. However, studies have shown that RGB-D fused input significantly improves grasp prediction performance. Therefore, both RGB and depth images are utilized in the system to achieve more accurate grasp estimation.
2. Feature Extraction and Output Generation: The grasp network extracts features from the preprocessed input and outputs three prediction maps: the grasp angle map, gripper width map, and grasp quality score map.
3. Grasp Inference: These three maps are further processed to infer the optimal grasp configuration, identifying the grasp point with the highest predicted quality.

## Proposed method

Kumra et al. [5] proposed the Generative Residual Convolutional Neural Network (GR-ConvNet), which predicts grasp configurations for unknown objects within a camera’s field of view. This network takes a depth image as input and outputs grasp pose estimations including grasp probability and grasp classification, aiming to predict the optimal grasp pose. Unlike traditional robotic grasp detection methods, GR-ConvNet generates three grasp-related maps—grasp angle, gripper width, and grasp quality—and infers the grasp rectangle by selecting the grasp with the highest probability from these maps. While GR-ConvNet is a classical method in robotic grasping, it suffers from suboptimal accuracy in grasp location and orientation. In this study, we improve upon GR-ConvNet by introducing CE-GR-NET, a Channel Exchange-based Generative Residual Convolutional Neural Network, designed to enhance grasp inference performance.

### Modality fusion module and method

We propose a modality fusion strategy that selectively exchanges channel features between the RGB and depth modalities to reduce redundancy and enhance relevant information. Specifically, we compare the scaling factors in the feature maps from both RGB and depth modalities against a predefined threshold. Channels in one modality with scaling factors below the threshold are replaced by corresponding channels from the other modality that exhibit higher scaling values. This operation effectively removes uninformative or redundant channels and enhances modality complementarity. The Batch Normalization (BN) layer, a widely used structure in neural networks, is employed to mitigate internal covariate shift and improve generalization performance. The transformation in a BN layer is defined as follows:


$$\begin{array}{c} x_{c}^{\prime }=\gamma_{c}\frac{x_{c}-\mu_{c}}{\sqrt{\sigma_{c}^{2}+\varepsilon }}+\beta_{c} \end{array}$$ (1)

here, $x_{c}^{\prime }$ and *x*_*c*_ represent the input and output feature maps of the c-th channel of the network, respectively. The terms $\mu_{c}$ and $\sigma_{c}$denote the mean and standard deviation of all activations at a given pixel position within the current mini-batch. The parameters $\beta_{c}$ and $\gamma_{c}$ are trainable bias and scaling factors, respectively. The constant ε is a small value added to prevent division by zero during normalization.

The scaling factor $\gamma_{c}$ in the Batch Normalization (BN) layer evaluates the correlation between *x*_*c*_ and $x_{c}^{\prime }$ during the training process. If $\gamma_{c}$ tends to zero during training, then the corresponding channel *x*_*c*_ will lose its influence on the final result. If the scaling factor of a channel falls below a slightly positive threshold during training, the channel becomes insignificant. Therefore, it is reasonable to replace the feature map of this channel with the corresponding channel from the other modality, as follows:


$$x{'}_{c}=\{\begin{array}{l} \gamma_{c}\frac{x_{c}-\mu_{c}}{\sqrt{\sigma_{c}^{2}+\varepsilon }}+\beta_{c},\text{if }\gamma_{c}>\text{threshold}\\ \gamma '{'}_{c}\frac{x'{'}_{c}-\mu '{'}_{c}}{\sqrt{\sigma '{'}_{c}^{2}+\varepsilon }}+\beta '{'}_{c},\text{else} \end{array}\;\;\;$$ (2)

here, $x_{c}^{\prime \prime }$, $\gamma_{c}^{\prime \prime }$, $\mu_{c}^{\prime \prime }$, $\sigma_{c}^{\prime \prime }$ and $\beta_{c}^{\prime \prime }$ represent the output feature map of the c-th channel in the other branch of the current path, as well as the associated parameters of the BN layer, respectively. The threshold γ is set as a small positive constant and determined empirically based on validation performance. In practice, the model is not highly sensitive to small variations of γ within a reasonable range, and a moderate value is selected to balance modality-specific feature preservation and cross-modal fusion.

The color-channel image and depth-channel image first undergo different convolution operations to obtain the same number of channels. After passing through the activation function, they are convolved with the same convolution kernel. Following normalization in the Batch Normalization (BN) layer, a channel exchange operation [21] is performed, where channels with larger scaling factors from the other modality replace the less important channels in the current modality. After the channel exchange, activation is applied. This channel exchange operation is performed twice. After the second channel exchange and activation, the feature maps from both the color modality and the depth modality are concatenated along the channel dimension. The resulting concatenated feature map is then passed through five residual layers, followed by three transposed convolution layers for the final output.

### CE-GR-NET-based grasping inference network

**Fig 4:**
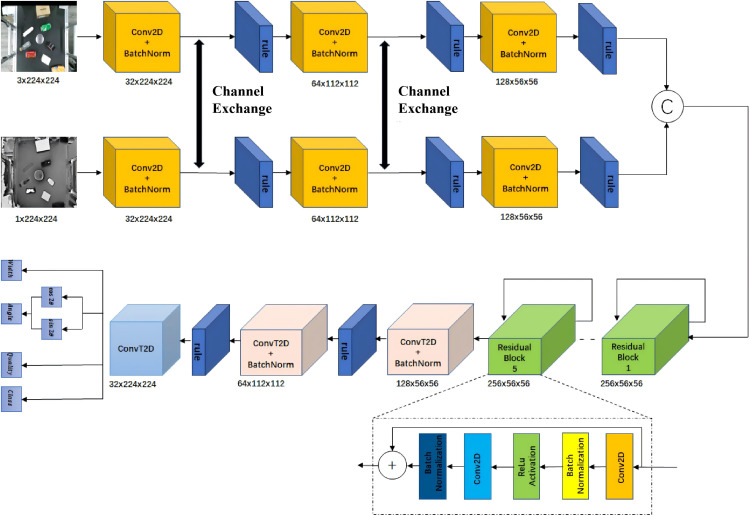
Architecture of the CE-GR-NET network.

The grasping inference network utilizes a Channel Exchange-Generative Residual Convolutional Neural Network (CE-GR-NET), as shown in Fig 4. The convolutional layers extract features from the input image and transform them through nonlinear operations to obtain high-level features. These outputs are then fed into five residual layers. In a neural network, each layer transforms the output of the previous layer. While increasing network depth can enhance feature representation, it simultaneously risks gradient vanishing; therefore, a residual architecture is employed to maintain stability. However, this transformation results in the compression and transformation of the input information, which may lead to information loss and gradient vanishing, making information flow difficult and resulting in the gradient vanishing problem that makes training challenging.In the residual layers, the output value is the sum of the input value of the previous layer and a residual term, which is the difference between the input and output of the previous layer. The introduction of residual layers helps the smooth flow of information through the network and mitigates the gradient vanishing issue. After passing through these convolutional and residual layers, the image size is reduced to 56×56. To facilitate the interpretation and preservation of spatial features after convolution operations, the image undergoes upsampling using transposed convolution, ensuring that the output image size matches the input image size of 224×224.

In the specific modality fusion module, two different branches receive the color-channel image and depth-channel image, respectively. On each branch: 1) Different convolution operations are applied to their respective input images to obtain the same number of channels, followed by activation; 2) The same convolution kernel is applied to perform convolutions, and after normalization in the BN layer, a channel exchange operation is performed. This operation replaces less important channels in the current branch with those having larger scaling factors from the other branch. Activation is applied after the channel exchange; 3) The same process of channel exchange is repeated for the second time. Finally, the feature maps from both branches are concatenated along the channel dimension. The concatenated feature map is then passed through five residual layers to alleviate the gradient vanishing problem, ensuring smoother information flow through the network. After the residual layers, a transposed convolution operation is applied to upsample the image, ensuring that the output image size matches the input image size.

To avoid redundant channels with scaling factors close to zero in both modalities, which could lead to a decrease in model capacity and make it difficult to determine the direction of channel exchange, we divide the channels into two parts. Channel exchange occurs only within the corresponding part. Additionally, we exploit the implicit sparsity induced by the scaling factors in the Batch Normalization (BN) layer to identify less informative channels and avoid ineffective channel exchanges. Specifically, channels with relatively small scaling factors are regarded as having low contribution and are selectively replaced during cross-modal fusion. The multi-task solid waste grasping inference network in this chapter takes solid waste scene images as input and outputs both the object categories and grasping configurations. The grasping representation is generated in terms of grasp center, grasp angle, gripper width, and grasp quality score. The proposed channel exchange algorithm is presented in Algorithm 1

**Algorithm 1. Channel Exchange at a Batch Normalization Layer**.

**Require:** Feature maps *x*_*m*_ for m∈{RGB,Depth} after a shared convolutional layer; BN parameters $\gamma_{m,c},\mu_{m,c},\sigma_{m,c},\beta_{m,c}$; threshold θ>0; channel partition ${𝒞}_{1},{𝒞}_{2}$ (disjoint halves of channel indices).

**Ensure:** Activated BN outputs ${\overset{\prime }{\underset{m,c}{\hat{x}}}}^{\prime }$ for each modality and channel.

1:  **for** each modality *m* and channel *c* **do**

2:   Compute normalized BN output: ${\hat{x}}_{m,c}=\gamma_{m,c}\cdot \frac{x_{m,c}-\mu_{m,c}}{\sqrt{\sigma_{m,c}^{2}+\epsilon }}+\beta_{m,c}$.

3:  **end for**

4:  **for** each channel $c\in {𝒞}_{1}$ **do**

5:   **if** $\gamma_{\text{RGB},c}<\theta$ and $\gamma_{\text{Depth},c}\geq \theta$ **then**

6:    $\overset{\prime }{\underset{\text{RGB},c}{\hat{x}}}\leftarrow {\hat{x}}_{\text{Depth},c}$ {Replace RGB output with Depth output}

7:   **else**

8:    $\overset{\prime }{\underset{\text{RGB},c}{\hat{x}}}\leftarrow {\hat{x}}_{\text{RGB},c}$

9:   **end if**

10:   $\overset{\prime }{\underset{\text{Depth},c}{\hat{x}}}\leftarrow {\hat{x}}_{\text{Depth},c}$ {Depth unchanged in ${𝒞}_{1}$}

11: **end for**

12: **for** each channel $c\in {𝒞}_{2}$ **do**

13:  **if** $\gamma_{\text{Depth},c}<\theta$ and $\gamma_{\text{RGB},c}\geq \theta$ **then**

14:   $\overset{\prime }{\underset{\text{Depth},c}{\hat{x}}}\leftarrow {\hat{x}}_{\text{RGB},c}$ {Replace Depth output with RGB output}

15:  **else**

16:   $\overset{\prime }{\underset{\text{Depth},c}{\hat{x}}}\leftarrow {\hat{x}}_{\text{Depth},c}$

17:  **end if**

18:  $\overset{\prime }{\underset{\text{RGB},c}{\hat{x}}}\leftarrow {\hat{x}}_{\text{RGB},c}$ {RGB unchanged in ${𝒞}_{2}$}

19: **end for**

20: **for** each modality *m* and channel *c* **do**

21:  ${\overset{\prime }{\underset{m,c}{\hat{x}}}}^{\prime }\leftarrow \text{ReLU}(\overset{\prime }{\underset{m,c}{\hat{x}}})$ {Apply activation after exchange}

22: **end for**

23: **return** ${\overset{\prime }{\underset{m,c}{\hat{x}}}}^{\prime }$ for m∈{RGB,Depth} and all channels *c*

### Generative inference module

The grasping posture representation is improved based on the grasp representation proposed by Morrison et al. [22], as shown in Equation 3:


$$\begin{array}{c} G_{r}=(P,\theta_{r},W_{r},Q,C) \end{array}$$ (3)

where *P*=(*x*,*y*,*z*) represents the center position of the gripper, $\theta_{r}$ is the angle of rotation of the gripper around the z-axis, *W*_*r*_ is the required width of the gripper, *Q* is the grasp quality score, and *C* is the object category classification

The network detects a grasp of height h and width w from an n-channel network image $I={\mathbb{R}}^{n\times h\times w}$, where the grasp can be defined as:


$$\begin{array}{c} G_{i}=(x,y,\theta_{i},W_{i},Q,C) \end{array}$$ (4)

where (*x*,*y*) corresponds to the grasp center in the image coordinates, $\theta_{i}$ represents the angle of rotation required to grasp the object at each pixel, denoted by the value of $\left[-\frac{\pi }{2},\frac{\pi }{2}\right]$, *W*_*i*_ is the required width at each pixel, represented as a value within the range [0, *W*_*max*_], and *W*_*max*_ is the actual maximum width of the gripper. The grasp quality score *Q* represents the grasp quality at each pixel in the image, with values ranging from 0 to 1, where a value closer to 1 indicates a higher likelihood of successful grasping. The object category *C* denotes the pixel-wise classification result, taking integer values in the range [0, 13], corresponding to the predefined object categories in the dataset. The detailed procedure of grasp candidate selection and filtering is described in Algorithm 2

To ensure consistency between pixel-wise predictions and object-level detection results, the outputs of the grasping network are integrated with the bounding boxes predicted by the object detection network (YOLOv5). Specifically, pixel-wise predictions are constrained within the detected object regions, and grasp candidates are selected only from pixels that lie inside the corresponding bounding boxes. This region-based filtering effectively reduces the impact of noisy pixel-wise labels and enforces consistency between detection and grasping.

**Algorithm 2. Grasp Candidate Selection with Detection Consistency**.

**Require:** RGB image *I*, grasp quality map *Q*, angle map Θ, width map *W*, category map *C*, detection boxes $\left\{B_{i}\right\}$ (from YOLOv5)

**Ensure:** Final grasp set $\left\{G_{i}\right\}$

1:  Run YOLOv5 to obtain detection boxes $\left\{B_{i}\right\}$

2:  **for** each detected object *B*_*i*_ **do**

3:   Extract region $Q_{i}=Q(B_{i})$

4:   Select candidate pixels where $Q_{i}>\tau$

5:   Apply non-maximum suppression (NMS) on *Q*_*i*_

6:   Select top-*k* candidates based on grasp quality

7:   **for** each candidate pixel **do**

8:    Retrieve θ, *w*, and *c* from Θ, *W*, *C*

9:    Generate grasp rectangle *G*

10:  **end for**

11: **end for**

12: **return** $\left\{G_{i}\right\}$

For a specific grasp rectangle, the evaluation criterion proposed by Redmon et al. [23] is adopted. A candidate grasp rectangle is considered valid only if it meets the following conditions: (1) The angle between the predicted grasp rectangle and the ground truth grasp rectangle is less than 30° and (2)The Intersection over Union (IoU) score between the predicted grasp rectangle *G*_*i*_ and the ground truth grasp rectangle ${\hat{G}}_{i}$ is greater than 0.25.


$$\begin{array}{c} IoU=\frac{\left|G\cap G^{\prime }\right|}{\left|G\cup G^{\prime }\right|}>0.25 \end{array}$$ (5)

For the dataset containing object $D=[D_{1},...,D_{n}]$, the input scene image $I=[I^{1},...,I^{n}]$ and the image frame $G_{i}=[g_{1}^{1},...,g_{m_{1}}^{1}...g_{1}^{2}...g_{m_{n}}^{1}]$ containing successful grasps are given. The model is trained by minimizing the negative log-likelihood of *I* conditioned on the input scene image *G*_*i*_, thereby learning the end-to-end mapping function $\gamma (I,D)=G_{i}$, which is defined as follows:


$$\begin{array}{c} -\frac{1}{n}\sum_{i=1}^{n}\frac{1}{m_{i}}\sum_{j=1}^{m_{i}}\log\gamma \left(g_{i}^{j}|I^{i}\right) \end{array}$$ (6)

The model uses the Adam optimizer [21] for parameter updates, with the standard backpropagation algorithm and mini-batch stochastic gradient descent technique employed during training. The learning rate is set to 10^−3^, and 8 samples are randomly selected for training in each iteration. A random seed is used during training to ensure consistent random splits across different training runs, eliminating variations introduced by randomness.

To mitigate the vanishing gradient problem, a smoothed Huber loss is used, and the CE-GR-NET network defines the loss as:


$$\begin{array}{c} \psi \left(G_{i},\hat{G_{i}}\right)=\frac{1}{n}\sum_{1}^{k}z_{k} \end{array}$$ (7)

where *z*_*k*_ is:


$$\begin{array}{c} z_{k}=\left\{ \begin{array}{cc} 0.5{\left(G_{i}-\hat{G_{i}}\right)}^{2}, & \text{if }\left|G_{i}-\hat{G_{i}}\right|<1\\ \left|G_{i}-\hat{G_{i}}\right|-0.5, & \text{otherwise} \end{array}\right. \end{array}$$ (8)

where *G*_*i*_ is the grasp configuration generated by the grasping network, and $\hat{G_{i}}$ is the ground truth grasp configuration.

The overall loss function is defined as a weighted combination of multiple task-specific losses:


$$\begin{array}{c} ℒ=\lambda_{q}{ℒ}_{quality}+\lambda_{\theta }{ℒ}_{angle}+\lambda_{w}{ℒ}_{width}+\lambda_{c}{ℒ}_{cls} \end{array}$$ (9)

where each term is computed using the smoothed Huber loss defined in Eq. (7)–(8). The weights $\lambda_{q}$, $\lambda_{\theta }$, $\lambda_{w}$, and $\lambda_{c}$ control the relative importance of each task.

The final grasping result will be represented as three images, where for each pixel, the grasping angle, grasping width, grasp quality score, and classification category are computed.

## Experiment

### Experimental data and details

#### Experimental datasets.

Comprehensive experiments were conducted on both the self-constructed dataset and the publicly available Cornell grasping dataset. The Cornell grasping dataset contains 1,035 RGB images with a resolution of 640×480 pixels and includes 240 different real-world objects. Importantly, the RGB images and corresponding depth maps in this dataset are pre-registered and spatially aligned at the pixel level, ensuring one-to-one correspondence between modalities. However, the Cornell dataset only provides grasp annotations on objects without specifying their categories. Therefore, additional category annotations were applied to the Cornell dataset to meet the input requirements of the YOLOv5 network. In practice, a total of 885 RGB images were used for object category annotation. The objects in the Cornell dataset were categorized into six major classes: tools, clothes, paper-box, bottle, foods, and others. The detailed annotation statistics are shown in Table 1. For both the Cornell dataset and the self-constructed dataset, RGB images and corresponding depth maps undergo identical preprocessing steps before being fed into the network. Specifically, both modalities are cropped, resized to 224×224, and normalized to ensure spatial consistency. For depth maps, invalid or missing values are handled using simple interpolation to maintain continuity. These preprocessing steps guarantee proper spatial alignment and consistency for subsequent feature-level fusion and channel exchange operations.

**Table 1:** Annotation Statistics of the Cornell Grasping Dataset.

Classes	tools	clothes	paper-box	bottle	foods	others
Number of Instances	389	86	28	161	103	118

#### Experimental setup.

Both the object detection network and the grasping network were trained under the same environment. The experiments were conducted on an Ubuntu 18.04.5 LTS system with Python 3.8.5, PyTorch 1.10.1, Torchvision 0.11.2, and CUDA 11.1. The hardware platform was equipped with an Intel Core i9-10920K CPU (3.50 GHz), an NVIDIA GeForce RTX 3090 GPU (24 GB), 64 GB of RAM, and a 2 TB SSD.

#### Evaluation metrics.

The following metrics were used to evaluate the model’s performance: True Positive (TP): the number of positive samples correctly identified by the classifier. False Positive (FP): the number of negative samples incorrectly predicted as positive. True Negative (TN): the number of negative samples correctly identified by the classifier. False Negative (FN): the number of positive samples incorrectly predicted as negative. The evaluation metrics used in this study include Precision (P) and Recall (R), which are calculated according to equations 10 and 11, respectively.


$$\begin{array}{c} P=\frac{TP}{TP+FP} \end{array}$$ (10)

$$\begin{array}{c} R=\frac{TP}{TP+FN} \end{array}$$ (11)

### Grasp network training and results

**Fig 5:**
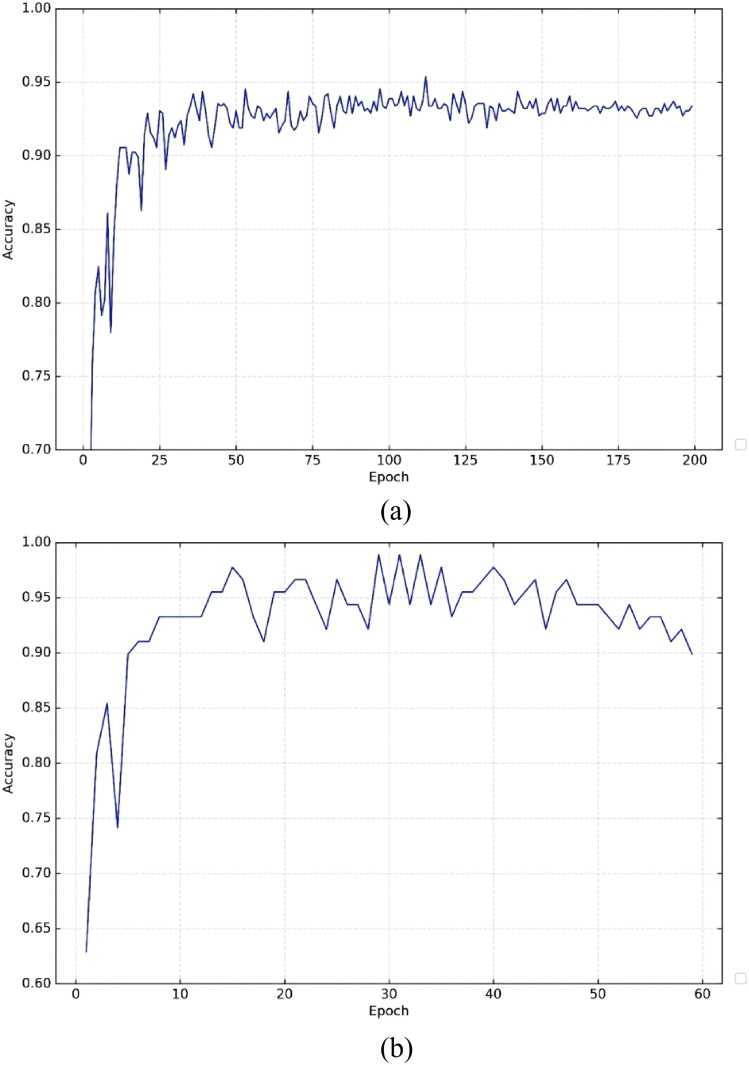
Training performance of the CE-GR-NET network: (a) Results on the Cornell Grasp Dataset; (b) Results on the custom-built dataset.

The grasp network training is evaluated using a rectangle-based metric. According to the proposed metric, a predicted grasp is considered valid if the Intersection over Union (IoU) between the predicted grasp rectangle and the ground-truth rectangle exceeds 25%, and the angular deviation between the predicted grasp orientation and the ground-truth is less than degrees. To convert pixel-wise grasp predictions into rectangular representations, the values of each pixel in the output image are mapped to their corresponding grasp rectangles. An epoch is defined as one complete pass through the entire dataset during training, followed by a weight update of the neural network. The number of training epochs is set to 60 on the Cornell Grasping Dataset, and to 200 on the custom dataset constructed in this study. The relationship between IoU values and the number of epochs is illustrated in Fig 5.

The network consists of approximately 1.9 million parameters, making it relatively lightweight compared to other grasping networks. As a result, it offers lower computational cost and faster inference speed than other grasp prediction architectures with millions of parameters and complex structures. A comparison of accuracy and inference speed on the Cornell Grasp Dataset between our network and other grasping networks is shown in Table 2. The results of the baseline methods are taken from their original publications under the standard Cornell evaluation protocol to ensure a fair and consistent comparison.

**Table 2:** Performance comparison of grasping networks on the Cornell Grasp Dataset.

Grasping Network	IW(%)	OW(%)	Grasp Inference Speed (ms)
Fast Search [17]	60.5	58.3	5000
SAE,struct.reg [24]	73.9	75.6	1350
MultiGrasp [22]	88.0	87.1	76
ResNet-50x2 [25]	89.2	88.9	103
GGCNN [16]	73.0	69.0	19
FCGN [26]	97.7	96.6	117
GraspNet [26]	90.2	90.6	24
GR-ConvNet [5]	97.7	96.6	20
SE-ResUNet [27]	98.2	97.1	25
SKGNET [28]	99.1	98.4	–
CE-GR-NET (ours)	**98.2**	**97.5**	**22**

IW represents randomly segmented images, and OW represents segmentation based on object instances. The bold values indicate the performance metrics corresponding to the method presented in this chapter.

### Object detection network training results

In this section, the object detection network is trained on the Cornell Grasping Dataset, and the results are shown in Table 3. Here, Precision represents the proportion of true positives in the predicted results, while Recall denotes the proportion of true positives that were correctly predicted from all actual positives. mAP50 refers to the mean average precision (mAP) calculated for each class with an Intersection over Union (IoU) threshold of 0.5, averaged across all images for each class, and mAP50-95 indicates the average mAP across different IoU thresholds (ranging from 0.5 to 0.95 with a step size of 0.05).

**Table 3:** Detection Results on the Cornell Grasping Dataset.

Classes	Precision	Recall	mAP50	mAP50-95
tools	0.993	0.997	0.994	0.895
clothes	0.991	0.997	0.995	0.938
paper-box	0.997	0.995	0.993	0.883
bottle	0.998	0.999	0.995	0.884
foods	0.994	1	0.991	0.895
others	0.996	0.998	0.991	0.846

**Fig 6:**
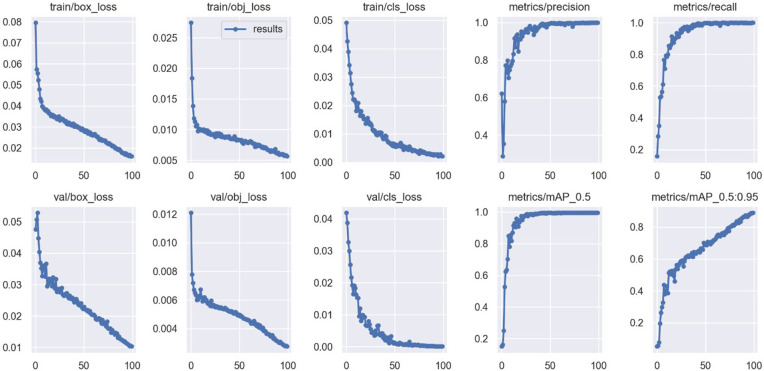
Training Results of the Object Detection Network.

As shown in Table 3, the object detection network achieves high precision, recall, and *mAP*@50 across the six object categories. One key reason for this performance is that the Cornell Grasping Dataset consists of single-object grasping targets with clear and distinctive visual features. The evaluation metrics over training epochs are illustrated in Fig 6, where the x-axis represents the number of training epochs and the y-axis corresponds to the metric values. Three loss components are used during training: box_loss, obj_loss, and cls_loss. Specifically, box_loss represents the localization loss, which measures the deviation between the predicted bounding boxes and the ground-truth boxes. obj_loss denotes the objectness loss, evaluating the overlap between predicted and ground-truth bounding boxes, indicating whether the predicted box covers an actual object. cls_loss refers to the classification loss, typically computed using a loss function that quantifies the difference between the predicted class and the true class label.

### Grasping experiment on custom dataset

In the dataset constructed for this study, multiple objects to be grasped may appear in a single image scene. In this case, the object detection network first performs detection based on the primary classification of the dataset. If any objects of interest are detected, their corresponding depth and color images are sent to the grasping detection network. The grasping detection network generates grasp configurations for all objects to be grasped in the image. When the grasp configuration center falls within the region of the object detection results, the corresponding grasp configuration is converted into a rectangular representation. The classification result of the grasping detection network is a secondary classification result, which is used for subsequent retrieval and processing.

It should be noted that the proposed method primarily relies on visual geometry derived from RGB-D data, and does not explicitly model physical properties such as surface friction, moisture, or material compliance, which may affect grasp success in real-world kitchen waste scenarios. However, the generative inference network learns grasp distributions from data in a data-driven manner, which implicitly captures certain object-specific grasping patterns related to their physical characteristics (e.g., stable grasp regions of bottles). Moreover, the network outputs multiple grasp candidates along with quality scores, allowing the system to select more reliable grasps under uncertainty. This design provides a degree of robustness compared to purely geometric or rule-based methods.

In terms of model accuracy, this study performs repeated training 5 times on the custom dataset, selecting the model with the highest grasping accuracy on the validation set as the base prediction model for subsequent steps. If multiple models achieve the same grasping accuracy, the one with the highest classification accuracy is chosen. On the custom dataset, the proposed method achieves an image-based model accuracy of 96.03%, an object-based model accuracy of 94.40%, and a grasped object classification accuracy of 97.87%. The average precision comparison between the proposed method and some generative grasping network models is shown in Table 4.

**Table 4:** Comparative Performance Analysis of CE-GR-NET against State-of-the-Art Models on the Cornell Dataset.

Average Precision Method	Image-Based Accuracy	Object-Based Accuracy	Classification Accuracy
GR-Convnet [5]	94.04%	93.48%	93.04%
SKGNet [27]	76.32%	93.54%	84.07%
SE-ResUnet [28]	95.03%	**94.58%**	95.82%
CE-GR-NET (ours)	**96.03%**	94.40%	**97.87%**

The bolded values represent the best performance results.

**Fig 7:**
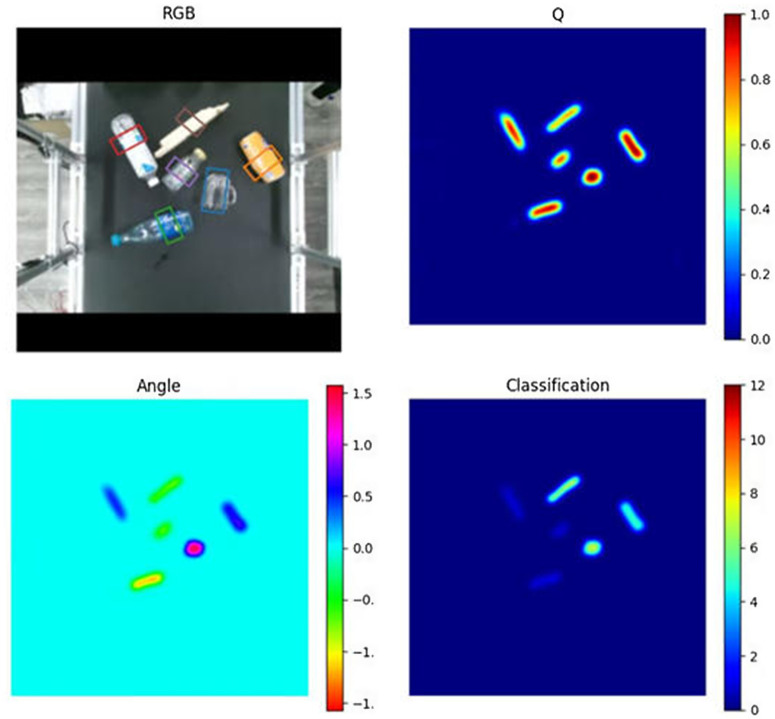
Inference Results on Multi-Source Urban Kitchen Waste Images.

According to the experimental results in Table 4, the proposed CE-GR-NET outperforms other methods in both image-based and classification-based grasping performance. Antipodal robotic grasping using generative residual convolutional neural network exhibits relatively weaker overall performance, primarily due to its generative residual architecture lacking the ability to effectively focus on grasp-relevant regions. The SKGNet (Selective Kernel convolution Grasp detection Network) method incorporates attention mechanisms and multi-scale feature fusion, enabling the network to better focus on grasping regions. While it performs well on public datasets such as Cornell, its performance degrades on the multi-source visual images used in this study due to the larger target objects and unstructured attention regions. The SE-ResUNet (Squeeze-and-Excitation ResUNet) method integrates residual blocks with channel attention mechanisms and demonstrates better performance in object-based grasping. However, its ability to fuse multi-scale information is inferior to the channel attention strategy adopted in our method. The inference results of the proposed method on multi-source kitchen waste images are shown in Fig 7.

## Conclusion

This study investigates a visual approach based on generative inference networks for grasp point generation of bottle-type waste in solid waste scenarios. By integrating an object detection network with a grasping network, the proposed framework enables accurate object identification and localization, while the channel-exchange-based generative residual inference network (CE-GR-NET) effectively generates grasping points. Experimental results on the Cornell Grasping Dataset demonstrate that the proposed method achieves reliable grasp inference in simple single-object scenarios with fast inference speed. Furthermore, experiments on the multi-source solid waste dataset constructed in this study show that the model can generate grasping rectangles while simultaneously associating them with corresponding object categories. Overall, the proposed CE-GR-NET framework demonstrates strong computational efficiency and good generalization capability across diverse waste categories, highlighting its potential for real-world robotic waste sorting applications.
